# The effect of strength training interventions on people with congenital heart disease: a systematic review

**DOI:** 10.1136/openhrt-2024-003091

**Published:** 2025-03-25

**Authors:** Kunyu Hao, Curtis Adrian Wadey, Alan R Barker, Craig A Williams

**Affiliations:** 1 Children's Health and Exercise Research Centre, Faculty of Health and Life Sciences, University of Exeter, Exeter, DEVON, UK; 2 Research and Innovation, Southern Health NHS Foundation Trust, Southampton, UK; 3 Research and Improvement, Hampshire and Isle of Wight Healthcare NHS Foundation Trust, Southampton, UK

**Keywords:** Cardiac Rehabilitation, Delivery of Health Care, Heart Defects, Congenital

## Abstract

**Aim:**

To assess the effectiveness and safety of strength training (ST) interventions in people with congenital heart disease (ConHD).

**Methods:**

Participants included people of all ages diagnosed with all complexity of ConHD. Interventions included strength training or inspiratory muscle training (IMT) which were delivered in whole or as part of a more holistic programme. Included studies were randomised controlled trials, non-randomised controlled trials and cohort studies. A comprehensive literature search using five databases until June 2023 was conducted. Two authors independently screened all the identified studies and assessed the risk of bias. Due to the paucity of studies and significant differences in study design, albatross plots were produced, and synthesis without meta-analyses was used to assist in the interpretation of results.

**Results:**

A total of 26 studies were included with a total of 659 participants (53% female). Three of five studies demonstrated that ST significantly improves muscle strength. The albatross plots (20 studies) compared peak oxygen consumption (peak V̇O_2_) of ST alone, combined training and IMT and showed that 16 studies observed an improvement with a standardised mean difference between 0.10 and 0.50. Combined training was more effective than ST alone and IMT for peak V̇O_2_, and ST alone was second. The results showed high heterogeneity. Three studies (one ST alone and two combined training) reported a total of five adverse events, but none reported serious adverse events or fatalities.

**Conclusion:**

This systematic review indicates a moderate improvement in muscle strength by ST alone, with a small improvement in peak V̇O_2_ in people with ConHD. Although the outcomes are positive, there is still insufficient evidence to establish the clinical significance of ST.

**WHAT IS ALREADY KNOWN ON THIS TOPIC**

- Physical activity can improve health-related fitness and quality of life in people with congenital heart disease (ConHD). Current exercise recommendations for cardiovascular disease advocate low-weight strength training (ST) as an adjunct to endurance training. Despite this acknowledgement, comprehensive recommendations regarding ST remain insufficiently addressed in exercise guidelines for people with ConHD.

**WHAT THIS STUDY ADDS**

- This review systematically examines the evidence on the effects of ST on muscle strength, cardiopulmonary fitness and pulmonary function in people with ConHD. The findings show that ST alone improves muscle strength, and both ST and combination training, as well as IMT, have a potential small improvement on peak V̇O_2_. The review highlights the safety of incorporating ST for individuals with ConHD.

**HOW THIS STUDY MIGHT AFFECT RESEARCH, PRACTICE OR POLICY**

- This systematic review comprehensively elucidated the effectiveness and safety of ST in people with ConHD to provide direction for prospective research endeavours and contribute data to support forthcoming healthcare and exercise management policies.

## Introduction

 Congenital heart disease (ConHD) includes over 20 types of structural heart and vessel abnormalities, occurring in about 1% of newborns worldwide.^1 2^ Advances in prenatal diagnosis and surgical correction have improved survival rates to 97% (2010–2017).^3^ As survival rates rise, the focus shifts to managing long-term health complications. Residual cardiopulmonary abnormalities such as diastolic function and chronotropic dysfunction after repair lead to impaired cardiopulmonary fitness (CRF).^4^ This limitation of CRF has increased risks of complications and reduced health-related fitness compared with healthy individuals, resulting in a lower quality of life.

Physical activity (PA) encompasses all forms of movement that improve health-related fitness and quality of life in ConHD patients.^5^ Strength training (ST), a subset of PA, involves muscle-strengthening exercises using various resistance forms, including body weight.^6^ Current resistance exercise recommendations for cardiovascular disease with good aerobic performance capacity and good left ventricular function advocate low-weight ST as an adjunct to endurance training; it can help positively influence psychosocial well-being and quality of life.^7^ An updated statement from the American Heart Association in 2023 emphasised the advantageous physiological and clinical impacts of ST in cardiovascular diseases and risk factors.^8^ Despite this acknowledgement, comprehensive recommendations regarding ST remain insufficiently addressed in exercise guidelines for people with ConHD.

Current studies of interventions involving ST for people with ConHD include ST alone, PA/exercise and inspiratory muscle training (IMT). In studies of PA interventions, low-weight ST (~30% maximum voluntary contraction) was included as part of PA.^9^,^11^ IMT, which aims to increase inspiratory muscle strength by targeting the diaphragm and intercostal muscles, may also be included in ST regimes.

The inclusion of ST in PA for individuals with ConHD is debated due to increased potential risks, such as increased blood pressure, reduced cardiac output and tachycardia.^12^ However, two recent studies have proposed that high-intensity ST increased CRF and muscle strength among Fontan patients,^13 14^ which casts doubt upon the prevailing notion advocating the avoidance of high-intensity ST for people with ConHD.^12^ Consequently, it is imperative to conduct a systematic review to comprehensively elucidate the effectiveness and safety of ST in people with ConHD to provide direction for prospective research endeavours and contribute data to support forthcoming healthcare and exercise management policies.

## Method

This systematic review aligns with the guidelines of Preferred Reporting Items for Systematic Reviews and Meta-Analyses. The protocol was registered with the International Prospective Register of Systematic Reviews (Registration no. CRD42023422423, https://www.crd.york.ac.uk/prospero/display_record.php?ID=CRD42023422423).

### Eligibility criteria 

This systematic review formulated eligibility criteria based on the PICOS (Participants, Interventions, Comparisons, Outcomes and Study Designs) framework. It comprised:

P: All people with ConHD, regardless of age and gender. 
I: The main interventions were strength, resistance, weight training or IMT, which were the whole or formed part of a wider intervention programme with a duration of 2 weeks or longer delivered in the hospital, in the gym or at home. These interventions included any structured, unstructured, multicomponent or single-component PA interventions. 
C: To compare with no-exercise and/or exercise control group or no comparator.  
O: No restriction was made in terms of intervention outcomes.
S: Randomised controlled trials (RCTs), non-randomised controlled trials (nRCTs) and cohort studies.  

Exclusion criteria:

1. The participants were not people with ConHD.
2. Strength, resistance, weight training or IMT was not included in the intervention.
3. The duration of the intervention was less than 2 weeks.

### Data collection

A comprehensive literature search was conducted using Medline, SPORTDiscus, Embase, Web of Science, Scopus and Cochrane Central from inception until June 2023. The search strategy followed the Peer Review of Electronic Search Strategy guidelines and was reviewed by an information specialist.^15^ The search terms consisted of keywords related to congenital heart disease and strength training and extended to include related Medical Subject Heading terms. Studies were limited to the English language. The full search strategy is available in online supplemental table 1.

Search results were screened using Rayyan software for duplicates and eligibility.^16^ Two review authors (KH and CW) independently screened titles and abstracts for inclusion from all the potential studies identified from the searches. The full-text version was read independently by two review authors (KH and CW) to confirm eligibility and document the reasons for exclusions. Two authors (CAW and ARB) arbitrated if any disagreements arose that could not be rectified through discussion. The search duration lasted from June 2023 to August 2023. Data extraction was done using Excel software, capturing study characteristics, intervention details and outcomes. The process was refined, and accuracy was checked through team discussions.

### Risk of bias in included studies

The risk of bias for CRF, muscle strength and pulmonary function in each study was assessed using the Risk of Bias V.2 tool for RCTs and the Risk of Bias in Nonrandomized Studies of Interventions tool for non-randomised studies (accessed: 15 November 2023).^17 18^ The first author performed the risk of bias assessment, and the accuracy of the assessment was subsequently verified by a second author through a random subsample (30%) of studies.

### Data synthesis 

Due to the lack of studies and differences in study design, the included studies were not available for conducting meta-analysis. To assist the interpretation of the results across studies, we produced albatross plots to peak V̇O_2_ and pulmonary function and undertook a synthesis without meta-analysis (SWiM)^19^ for muscle strength and adverse events. Albatross plots, which require only the p value, standardised mean difference (SMD) of pre-post changes and total sample size, provide an approximate effect size examination and identify heterogeneity sources.^20^ We extracted the results pre-post intervention from the included studies. If only the mean and CIs were provided, the SD was calculated according to the Cochrane Handbook.^21^ The p value and the total sample size were based on the mean, SD and sample size. The SMD was calculated to compare the average pre-post response to reflect the effect size, which was reflected in the contour line of the albatross plot, with SMD values of small (0.1–0.49), medium (0.5–0.79) and large (≥0.8).^22^ Subgroup analyses were performed according to different interventions (ST, IMT and combined training), age of participants (paediatrics and adults) and types of ConHD (Fontan, tetralogy of Fallot (ToF) and mixture). Considering that the mean age of participants in the study ranged from 12 to 41 years, we categorised the age group into paediatrics (12–18 years) and adults (>18 years).

### Summary of findings

We employed GRADEpro Guideline Development Tool (GDT)^23^ to construct a ‘Summary of findings’ to evaluate the certainty of the evidence, encompassing the following outcomes: muscle strength, CRF, pulmonary function and adverse events.

## Results

### Study selection

**Figure 1:**
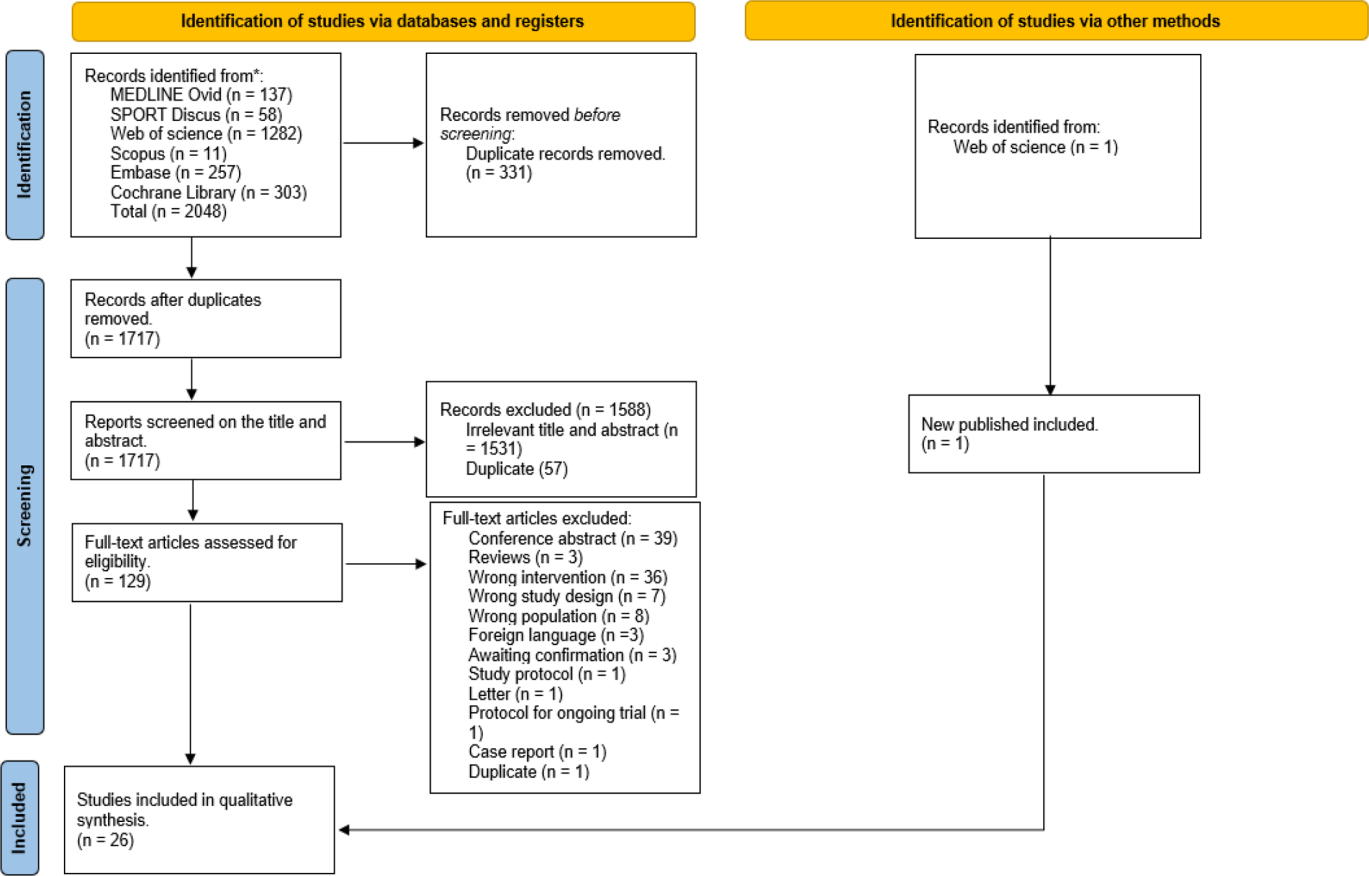
Preferred Reporting Items for Systematic Reviews and Meta-Analyses flow chart of the studies.

Figure 1 illustrates the flow chart of the study selection process. Initially, 2048 potential studies were identified. Of the 129 studies assessed as full text, 25 studies fulfilled the criteria for eligibility and were included in the review. During the search, email communication with an author revealed a new article fitting the criteria, which was included after its publication on 5 September 2023.^14^ Consequently, a total of 26 studies were included.

### Study design and participants

Table 1 presents the study population and design of the included studies. A total of 654 people with ConHD (338 males and 316 females) and 21 healthy controls participated. 8 studies were RCTs,^1424^,^30^ 8 were nRCTs^1011 13 31^,^35^ and 10 were cohort studies.^936^,^44^ The ages of the participants ranged from 12 to 41 years, with 14 studies focusing on paediatrics (12–18 years),^9^,^1114 24 30 33 34 36 38 40^ and 12 for adults (> 18 years).^1325^,^29 31 32 35 37 39 43^

**Table 1:** Characteristics of the included studies

Study	Year	Age (mean)	Age(SD/range)	Sex (% male)	Country	CHD diagnosis (n) (% ConHD)	Training group (n)	Control group (n)	Drop out	Study design
**Strength training**										
Scheffer *et al*^14^	2023	12.9 y	10.5–15.7	17 M, 10 F (63%)	Netherlands	Fontan (40)	27	14 (ConHD)	1	RCT
Shiina *et al*^29^	2019	40.3 y	8.91	10 M, 15 F (40%)	Japan	Training group: ToF/DORV (11), Fontan (2), cyanosis (2)Control group: ToF/DORV (9), Fontan (3), cyanosis (3)	15	15 (ConHD)	0	RCT
Cordina *et al*^13^	2013	32 y	2	9 M, 2 F (82%)	Australia	Fontan (5)	6	5 (health)	5	nRCT
Lahti *et al*^34^	2022	12.53 y	2.19	9 M, 10 F (47.4%)	Canada	Fontan (10)	10	9 (health)	7	nRCT
Avitabile *et al*^41^	2022	15.6 y	1.7	10 M, 10 F (50%)	USA	Fontan (20)	20	NR	9	Cohort study
**IMT**										
Turquetto *et al*^27^	2021	20 y	15–25	5 M, 17 F (77%)	Brazil	Fontan (22)	10	12 (ConHD)	10	RCT
Hock *et al*^24^	2022	15.25 y	4.87	27 M, 23 F (61.7%)	Germany	ToF (30)	30	30 (ConHD)	6	RCT
Fritz *et al*^25^	2020	28.6 y	24.7–36.5	21 M, 21 F (50%)	Germany	Fontan (42)	20	22 (ConHD)	9	RCT
Neidenbah *et al*^30^	2023	12.4 y	1.9	30 M, 10 F (75%)	Germany	Fontan (40)	20	20 (ConHD)	3	RCT
Brown *et al*^31^	2020	34.21 y	13.97	5 M, 23 F (77%)	UK	ConHD complexity (28)	10	18 (ConHD)	5	nRCT
Laohachai *et al*^42^	2017	16 y	2	12 M, 11 F (52%)	Australia	Fontan (23)	23	NR	4	Cohort study
Wu *et al*^43^	2018	28.8 y	7.8	6 M, 5 F (55%)	USA	Fontan (11)	11	NR	1	Cohort study
**Combined training**										
Opotowsky *et al*^28^	2018	41.1 y	12.1	14 M, 14 F (50%)	America	ToF (13), SRV (9), Fontan (2), PA (2), TA (1), EA (1)	13	15 (ConHD)	5	RCT
Avila *et al*^26^	2016	35 y	28–42	11 M, 6 F (65%)	Canada	ToF (17)	13	4 (ConHD)	0	RCT
Rhodes *et al*^11^	2006	12.01 y	2.27	25 M, 8 F (75.8%)	USA	Training group: Fontan (11), Senning (1), DORV/PS (1), Ebstein (1), VSD/PS (1)Control group: Fontan (15), ToF (2), DORV (1), VSD/PS (1)	15	18 (ConHD)	NR	nRCT
Singh *et al*^10^	2007	12.1 y	1.8	NA	USA	Training group: Fontan (11), other (3)Control group: Fontan (13), other (2)	14	15 (ConHD)	NR	nRCT
Brassard *et al*^33^	2006	17.5 y	6.08	8 M, 6 F (58%)	Canada	Fontan (7)	7	7 (Health)	2	nRCT
Martinez *et al*^35^	2010	27.7 y	8.5	5 M, 3 F (62.5%)	Spain	VSD (4), DORV (1), PA+BT (1), UV (1), CAVSD (1)	4	4 (ConHD)	NR	nRCT
Gierat-Haponiuk *et al*^32^	2015	23 y	3.4	27 M, 30 F (47.3%)	Poland	VSD (30), ASD (27)	31	26 (ConHD)	0	nRCT
Sutherland *et al*^36^	2018	15 y	12–19	10 M, 7 F (59%)	Australia	Fontan (17)	17	NR	2	Cohort study
Aronoff *et al*^39^	2023	19 y	7.3	23 M, 24 F (49%)	USA	Fontan (9), ToF (5), AVSD (3), D-TGA (3), EA (2), Glenn (1),	47	NR	26	Cohort study
Fernie *et al*^40^	2023	13.5 y	3	21 M, 24 F (42.9%)	USA	Fontan (9)	9	NR	17	Cohort study
Becker-Grunig *et al*^37^	2013	48 y	11	4 M, 16 F (20%)	Germany	ConHD-APAH (20)	20	NR	0	Cohort study
Ferrer-Sargues *et al*^9^	2020	14.4 y	1.1	9 M, 6 F (60%)	Spain	ToF (6), D-TGA (2), VSD (2) (67%)	15	NR	0	Cohort study
Pyykkonen *et al*^44^	2022	14.5 y	2.6	10 M, 6 F (62.5%)	Finland	Fontan (16)	16	NR	2	Cohort study
Dirks *et al*^38^	2022	16.5 y	10–43	10 M, 8 F (55.6%)	Germany	Fontan (18)	18	NR	0	Cohort study

ASD, atrial septal defect; AVSD, atrioventricular septal defect; ConHD, congenital heart disease; ConHD-APAH, congenital heart disease-associated pulmonary arterial hypertension; DORV, double outlet right ventricle; D-TGA, dextro-transposition of the great arteries; EA, Ebstein’s anomaly; F, female; IMT, inspiratory muscle training; M, male; nRCT, non-randomised controlled trials; PS, pulmonary valve stenosis; RCT, randomised controlled trials; SRV, systemic right ventricle; ToF, tetralogy of Fallot; VSD, ventricular septal defect; y, year.

Regarding ST interventions, five studies used ST alone,^13 14 29 34 41^ seven used only IMT^24 25 27 30 31 42 43^ and 14 studies incorporated combined training, where ST was delivered as part of a wider programme (table 2). 12 studies lasted 12 weeks,^9^,^1114 26 28 31 34 35 37 39 43^ with ST alone and combined training conducted two to three times per week, and IMT performed five to seven times per week, each session lasting 30–60 min. In combined training, ST accounted for 34% of the total training time (15–40 min). Four studies used repetition maximum to measure ST intensity,^9 13 14 29^ and six studies gradually increased intensity.^9 14 26 34 36 40 41^ Combined training generally involved low-intensity ST with lighter weights (~1–2 kg) and lacked standardisation. The intensity of IMT was primarily measured by maximum inspiratory pressure percentage. Six studies did not report training intensity.^10 11 28 30 31 44^

**Table 2:** Type, frequency, duration, intensity, time and location of intervention of the included studies

Study	Year	Type of intervention (strength, IMT, aerobic or in combination)	Frequency (times per week)	Duration (number of weeks)	Intensity	Time(length per session in minutes)	Location of training
**Strength training**							
Scheffer *et al*^14^	2023	ST	3	12	6–8RM	45 (total)	Supervised
Shiina *et al*^29^	2019	ST+amino acid intake	3	8	15RM	NR	Home
Cordina *et al*^13^	2013	ST	3	20	8RM	60 (total)	Supervised
Lahti *et al*^34^	2022	ST	3	12	4–6 RPE	30–45 (total)	Supervised and home
Avitabile *et al*^41^	2022	ST	2	24	60% MVC	60 (total)	Supervised and home
**IMT**							
Turquetto *et al*^27^	2021	Aerobic/IMT	3 (aerobic) 7 (IMT)	16	NR (aerobic), 60% MIP (IMT)	60 (aerobic), NR (IMT)	Supervised and home
Hock *et al*^24^	2022	IMT	7	24	40% FVC	1–3 sets with 10–30 repetitions	Home
Fritz *et al*^25^	2020	IMT	7	48	Adjusted until maximum	3 sets with 10–30 repetitions	Home
Neidenbah *et al*^30^	2023	IMT	7	24	NR	NR	Supervised and home
Brown *et al*^31^	2020	IMT	3	12	NR	NR	Home
Laohachai *et al*^42^	2017	IMT	7	6	30% MIP	30 (total)	Home
Wu *et al*^43^	2018	IMT	5	12	40% MIP	30 (total)	Supervised
**Combined training**							
Opotowsky *et al*^28^	2018	Aerobic+ST	2	12	NR	60 (total)	Supervised and home
Avila *et al*^26^	2016	Aerobic+ST	3 (aerobic), 2 (ST)	12	70%–80% hour	60 (total), 30–40 (ST)	Supervised
Rhodes *et al*^11^	2006	Aerobic+ST	2	12	NR (aerobic), light weight (ST)	60 (total), 20–25 (ST)	Home
Singh *et al*^10^	2007	Aerobic+ST	2	12	NR (aerobic), light weight (ST)	60 (total), 20–25 (ST)	Home
Brassard *et al*^33^	2006	Aerobic+ST	3	8	50%–80% peak V̇O_2_, 12–15RM (SR)	60 (total)	Supervised and home
Martinez *et al*^35^	2010	Aerobic+ST	2	12	80% hour (Aerobic), 1–2 kg (ST)	60 (total), 10 (ST)	Supervised
Gierat *et al*^32^	2015	Aerobic+ST	2–3	4	60%–80% hour, 15 RM (ST)	60 (total)	Supervised
Sutherland *et al*^36^	2018	Aerobic+ST	2	8	65%–85% hour (Aerobic), Body weight (ST)	60 (total), 20–30 (ST)	Supervised and home
Aronoff *et al*^39^	2023	Aerobic+ST	2–3	12	70%–80% hour, Light weight (ST)	60 (total), 15 (ST)	Supervised and home
Fernie *et al*^40^	2023	Aerobic+ST	3–4	48	Reserve HR	60 (total)	Supervised
Becker-Grunig *et al*^37^	2013	Aerobic+ST＋IMT	7 (hospital)+5 (home)	3 (hospital)+12 (home)	60%–80% hour (aerobic)500 g–1 kg (ST), NR (IMT)	90 (hospital), 30 (home)	Supervised and home
Ferrer *et al*^9^	2020	Aerobic+ST + IMT	2	12	75% of HR (aerobic)10–15 RM (ST), 30% MIP (IMT)	70 (total)20 (ST)	Supervised
Pyykkonen *et al*^44^	2022	Aerobic+ST	2	24	NR	NR	Home
Dirks *et al*^38^	2022	Aerobic+IMT	6/7 (IMT), 3 (aerobic)	40	30–50% of MIP (IMT)55% of W at V̇O_2_ max (aerobic)	1 set with 30 repetitions (IMT)45 min (aerobic)	Home

If CON data are absent in the respective study, then not reported

FVC, forced vital capacity; HR, heart rate; IMT, inspiratory muscle training; MEP, maximal expiratory pressure; MIP, maximal inspiratory pressure; MVC, maximum voluntary contraction; NR, not reported; RM, repetition maximum; ST, strength training.

### Outcomes

#### Muscle strength

Muscle strength outcomes from five studies are reported using SWiM (table 3), with three studies demonstrating significant improvements.^13 14 27 31 33^ Scheffer *et al*^14^ documented muscle strength assessments using maximum voluntary contraction (MVC) across muscle groups among young Fontan patients, all of which reported statistically significant increases in strength. Cordina *et al*^13^ reported that 20 weeks of high-intensity ST increased muscle strength in people with ConHD. Turquetto *et al*^27^ observed no change in grip strength following IMT intervention compared with controls, whereas Brown *et al*^31^ reported improvements in grip, biceps and quadriceps strength after IMT intervention. Brassard *et al*^33^ reported no significant strength increase in biceps and quadriceps with combined training (aerobic training and ST).

**Table 3:** The results of the cardiopulmonary exercise test, pulmonary function, muscle function and adverse outcome of the included studies

Study	Year	CPET values	
		Before	After
**Strength training**			
Cordina *et al*^13^	2013	ConHD: peak ˙V̇O_2_ (change): 183±3.1 (mL/min)Control: peak ˙V̇O_2_ (change): 5±39 (mL/min)	
Lahti *et al*^34^	2022	ConHD: V̇O_2_ reserve: 27.2±5.7 (mL/kg/min), peak HR: 175±23 (beats per minute)	ConHD: V̇O_2_ reserve: 29.4±8.8 (mL/kg/min), peak HR: 169±21 (beats per minute)
Avitabile *et al*^41^	2022	ConHD: peak ˙V̇O_2_: 28.43±5.55 (mL/kg/min), VE/VCO_2_: 34.7±3.6 (slope)ConHD: peak HR: 174.65±10.33 (beats per minute), peak work rate: 121.9±29.82 (W)	ConHD: peak ˙V̇O_2_: 28.93±5.58 (mL/kg/min), VE/VCO_2_: 34.6±4.89 (slope)ConHD: peak HR: 176.8±9.25 (beats per minute), peak work rate: 131.25±35.12 (W)
Scheffer *et al*^14^	2023	ConHD: peak V̇O_2_: 33.3 (mL/kg/min), peak HR: 160 (beats per minute), peak work rate: 122 (W)Control: peak V̇O_2_: 35.2 (mL/kg/min), peak HR: 166 (beats per minute), peak work rate: 96.5 (W)	ConHD: peak V̇O_2_: 35.5 (mL/kg/min), peak HR: 163 (beats per minute), peak work rate: 129 (W)Control: peak V̇O_2_: 33.3 (mL/kg/min), peak HR: 151 (beats per minute), peak work rate: 88 (W)
**IMT**			
Turquetto *et al*^27^	2021	ConHD: peak V̇O_2_: 26.6 (mL/kg/min), peak HR: 175 (beats per minute), V̇E/V̇CO_2_: 36 (slope)Control: peak V̇O_2_: 29.7 (mL/kg/min), peak HR: 173 (beats per minute), VE/VCO_2_: 31 (slope)	ConHD: peak V̇O_2_: 29.1 (mL/kg/min), peak HR: 176 (beats per minute), V̇E/V̇CO_2_: 36 (slope)Control: peak V̇O_2_: 27.6 (mL/kg/min), peak HR: 174 (beats per minute), VE/VCO_2_: 31 (slope)
Hock *et al*^24^	2022	ConHD: peak V̇O_2_: 31.1±6.5 (mL/kg/min), VE/VCO_2_: 30.2±4.3 (slope)Control: peak V̇O_2_: 31.4±7.6 (mL/kg/min), V̇E/V̇CO_2_: 29.6±3.6 (slope)	ConHD: peak V̇O_2_: 31.6±7.3 (mL/kg/min), VE/VCO_2_: 30±4.3 (slope)Control: peak V̇O_2_: 29±6.5 (mL/kg/min), V̇E/V̇CO_2_: 30±4.5 (slope)
Fritz *et al*^25^	2020	ConHD: peak V̇O_2_: 23.4 (mL/kg/min), peak HR: 148.5 (beats per minute), V̇E/V̇CO_2_: 31.3 (slope)Control: peak V̇O_2_: 24.1 (mL/kg/min), peak HR: 152.5 (beats per minute), V̇E/V̇CO_2_: 33.4 (slope)	ConHD: peak V̇O_2_: 23.3 (mL/kg/min), peak HR: 142 (beats per minute), V̇E/V̇CO_2_: 31.4 (slope)Control: peak V̇O_2_: 22.5 (mL/kg/min), peak HR: 158.5 (beats per minute), V̇E/V̇CO_2_: 33.5 (slope)
Neidenbah *et al*^30^	2023	ConHD: peak V̇O_2_: 35.74±6.89 (mL/kg/min), peak HR: 171.95±17.37 (beats per minute)ConHD: peak work rate: 123.89±46.36 (W), V̇E/V̇CO_2_: 32.05±3.75 (slope)Control: peak V̇O_2_: 32.98±5.84 (mL/kg/min), peak HR: 168.05±20.39 (beats per minute)Control: peak work rate: 111.32±48.23 (W), V̇E/V̇CO_2_: 32.4±3.95 (slope)	ConHD: peak V̇O_2_: 36.76±7.84 (mL/kg/min), peak HR: 171±11.83 (beats per minute)ConHD: peak work rate: 140.44±42.54 (W), V̇E/V̇CO_2_: 31.43±3.5 (slope)Control: peak V̇O_2_: 33.93±6.35 (mL/kg/min), peak HR: 170.11±15.74 (beats per minute)Control: peak work rate: 118.5±49.17 (W), V̇E/V̇CO_2_: 32.04±2.89 (slope)
Laohachai *et al*^42^	2017	ConHD: peak V̇O_2_: 26.8±6.8 (mL/kg/min), V̇E/V̇CO_2_: 34.2±7.8 (slope)ConHD: peak HR: 174±15 (beats per minute), peak work rate: 120±28 (W)	ConHD: peak V̇O_2_: 26±7.2 (mL/kg/min), V̇E/V̇CO_2_: 32.2±5.6 (slope)ConHD: peak HR: 171±19 (beats per minute), peak work rate: 118±28 (W)
Wu *et al*^43^	2018	ConHD: peak V̇O_2_: 22.2±8.0 (mL/kg/min), peak HR: 130.7±33.1 (beats per minute)ConHD: peak work rate: 116.5±45 (W), V̇E/V̇CO_2_: 34.1±6.7 (slope)	ConHD: peak V̇O_2_: 24±9.8 (mL/kg/min), peak HR: 134.9±32.1 (beats per minute)ConHD: peak work rate: 126.8±47 (W), V̇E/V̇CO_2_: 31.4±3.6 (slope)
**Combined training**			
Opotowsky *et al*^28^	2018	ConHD: peak V̇O_2_: 15.4±3.5 (mL/kg/min), peak work rate: 93.6±21.7 (W)ConHD: V̇E/V̇CO_2_: 32.9±5.1 (slope)Control: peak V̇O_2_: 18±3.8 (mL/kg/min), peak work rate: 111.32±48.23 (W)Control: V̇E/V̇CO_2_: 30.9±6.1 (slope)	N/A
Avila *et al*^26^	2016	ConHD: peak V̇O_2_: 26.2 (mL/kg/min), peak HR: 171 (beats per minute)Control: peak V̇O_2_: 28.1 (mL/kg/min), peak HR: 177 (beats per minute)	ConHD: peak V̇O_2_: 27.1 (mL/kg/min), peak HR: 171 (beats per minute)Control: peak V̇O_2_: 29 (mL/kg/min), peak HR: 168 (beats per minute)
Rhodes *et al*^11^	2006	ConHD: peak V̇O_2_: 24.8±6.8 (mL/kg/min), V̇E/V̇CO_2_: 36.1±5.3 (slope)ConHD: peak HR: 158±24 (beats per minute)Control: peak V̇O_2_: 27.9±5.6 (mL/kg/min), V̇E/V̇CO_2_: 34.8±6.1 (slope)Control: peak HR: 155±17 (beats per minute)	ConHD: peak V̇O_2_: 30.2±7.5 (mL/kg/min), V̇E/V̇CO_2_: 39.5±10.3 (slope)ConHD: peak HR: 162±27 (beats per minute)Control: N/A
Singh *et al*^10^	2007	ConHD: peak V̇O_2_: 26.3±9.6 (mL/kg/min), peak HR: 159±25 (beats per minute)Control: peak V̇O_2_: 27.6±5.6 (mL/kg/min), peak HR: 159±18 (beats per minute)	ConHD: peak V̇O_2_: 31.2±7.5 (mL/kg/min), peak HR: 164±26 (beats per minute)Control: peak V̇O_2_: 27.9±6.1 (mL/kg/min), peak HR: 156±17 (beats per minute)
Brassard *et al*^33^	2006	ConHD: peak V̇O_2_: 26.8±3.0 (mL/kg/min), V̇E/V̇CO_2_: 41.8±3.5 (slope)ConHD: peak HR: 152±14 (beats per minute), peak work rate: 115±20 (W)	ConHD: peak V̇O_2_: 25.9±3.1 (mL/kg/min), V̇E/V̇CO_2_: 40.7±3.7 (slope)ConHD: peak HR: 150±12 (beats per minute), peak work rate: 114±19 (W)
Martinez *et al*^35^	2010	ConHD: peak HR: 106.5±29.8 (beats per minute)Control: peak HR: 82.5±20.2 (beats per minute)	ConHD: peak HR: 98.7±12.1 (beats per minute)Control: peak HR: 95.5±20.9 (beats per minute)
Gierat-Haponiuk *et al*^32^	2015	ConHD: peak V̇O_2_: 27.5±3.5 (mL/kg/min), peak HR: 163±13 (beats per minute)ConHD: peak work rate: 144±18.2 (W)Control: peak V̇O_2_: 23±5.8 (mL/kg/min), peak HR: 152±23 (beats per minute)Control: peak work rate: 124±39.4 (W)	ConHD: peak V̇O_2_: 23.5±3.4 (mL/kg/min), peak HR: 143±18 (beats per minute)ConHD: peak work rate: 118±24.7 (W)Control: peak V̇O_2_: 23±6.0 (mL/kg/min), peak HR: 148±25 (beats per minute)Control: peak work rate: 122.5±36 (W)
Sutherland *et al*^36^	2018	ConHD: peak V̇O_2_: 24.6±6.2 (mL/kg/min), peak HR: 173±14 (beats per minute)ConHD: peak work rate: 118±44 (W)	ConHD: peak V̇O_2_: 25.9±7.1 (mL/kg/min), peak HR: 167±13 (beats per minute)ConHD: peak work rate: 121±42 (W)
Aronoff *et al*^39^	2023	ConHD: peak V̇O_2_: 25.6±7.8 (mL/kg/min), V̇E/V̇CO_2_: 32.5±8.2 (slope)ConHD: peak HR: 157.4±28.4 (beats per minute)	ConHD: peak V̇O_2_: 22.6±7.6 (mL/kg/min), V̇E/V̇CO_2_: 34±8.5 (slope)ConHD: peak HR: 157.4±28.7 (beats per minute)
Fernie *et al*^40^	2023	ConHD: peak ˙V̇O_2_: 28.8±8.5 (mL/kg/min), V̇E/V̇CO_2_: 32.7±4.7 (slope)ConHD: peak HR: 172±16 (beats per minute), peak work rate: 143.3±44.1 (W)	N/A
Becker-Grunig *et al*^37^	2013	ConHD: peak V̇O2: 11.4±2.2 (mL/kg/min), peak HR: 119±18 (beats per minute)ConHD: peak workload: 58±20 (W)	ConHD: peak V̇O2: 12.3±2.4 (mL/kg/min), peak HR: 138±15 (beats per minute)ConHD: peak workload: 75±23 (W)
Pyykkonen Henri *et al*^44^	2022	ConHD: peak V̇O_2_: 28±5.9 (mL/kg/min), peak HR: 167±16 (beats per minute)ConHD: peak work rate: 119±39 (W), V̇E/V̇CO_2_: 32.8±7.6 (slope)	ConHD: peak V̇O_2_: 29±7.2 (mL/kg/min), peak HR: 169±15 (beats per minute)ConHD: peak work rate: 132±44 (W), V̇E/V̇CO_2_: 30.0±5.0 (slope)
Dirks *et al*^38^	2022	ConHD: peak V̇O_2_: 26.5±2.1 (mL/kg/min), V̇E/V̇CO_2_: 33.02±1.32 (slope)	ConHD: peak V̇O_2_: 28.1±1.95 (mL/kg/min), V̇E/V̇CO_2_: 32.51±1.45 (slope)
		**Pulmonary function**	
		**Before**	**After**
**IMT**			
Turquetto *et al*^27^	2021	ConHD: FVC: 2.2 (L), FEV1: 2 (L)Control: FVC: 2.7 (L), FEV1: 2.3 (L)	ConHD: FVC: 2.5 (L), FEV1: 2.2 (L)Control: FVC: 2.7 (L), FEV1: 2.3 (L)
Hock *et al*^24^	2022	ConHD: FVC: 2.9±1.0 (L), FEV1: 2.5±0.9 (L), FEV1/FVC: 87.3±6.2 (%)Control: FVC: 3.0±0.8 (L), FEV1: 2.6±0.7 (L) FEV1/FVC: 86.7±5.8 (%)	ConHD: FVC: 3.0±1.1 (L), FEV1: 2.6±0.9 (L), FEV1/FVC: 86.6±5.5 (%)Control: FVC: 3.0±0.8 (L), FEV1: 2.5±0.8 (L), FEV1/FVC: 83.5±6.6 (%)
Fritz *et al*^25^	2020	ConHD: FVC: 3.8 (L), FEV1: 3.1 (L), FEV1/FVC: 80.6 (%)Control: FVC: 3.6 (L), FEV1: 3.0 (L), FEV1/FVC: 82.8 (%)	ConHD: FVC: 3.8 (L), FEV1: 3.0 (L), FEV1/FVC: 77.7 (%)Control: FVC: 3.6 (L), FEV1: 3.0 (L), FEV1/FVC: 81.5 (%)
Neidenbah *et al*^30^	2023	ConHD: FVC: 2.58±0.75 (L), FEV1: 2.25±0.67 (L), FEV1/FVC: 87.26±7.13 (%)Control: FVC: 2.3±0.79 (L), FEV1: 2.1±0.73 (L), FEV1/FVC: 91.36±6.07 (%)	ConHD: FVC: 2.84±0.75 (L), FEV1: 2.45±0.71 (L), FEV1/FVC: 86.13±9.41 (%)Control: FVC: 2.57±0.87 (L), FEV1: 2.28±0.78 (L), FEV1/FVC: 89.3±7.18 (%)
Laohachai *et al*^42^	2017	ConHD: FVC: 3.3±0.8 (L), FEV1: 2.9±0.7 (L), FEV1/FVC: 87.8±5.6 (%)	ConHD: FVC: 3.3±0.8 (L), FEV1: 2.9±0.7 (L), FEV1/FVC: 83.3±7.5 (%)
		**Muscle strength**	
		**Before**	**After**
**Strength training**			
Scheffer *et al*^14^	2023	ConHD: shoulder abduction: 120 (N), elbow flexion: 165 (N), elbow extension: 97 (N)ConHD: hip flexion: 221 (N), hip abduction: 137 (N), knee flexion: 141 (N)ConHD: knee extension: 144 (N)	ConHD: shoulder abduction: 132 (N), elbow flexion: 177 (N), elbow extension: 103 (N)ConHD: hip flexion: 300 (N), hip abduction: 202 (N), knee flexion: 157 (N)ConHD: knee extension: 195 (N)
Cordina *et al*^13^	2013	Muscle strength increased 43%	
Turquetto *et al*^27^	2021	ConHD: handgrip strength: 18 (kgf)Control: handgrip strength: 19 (kgf)	ConHD: handgrip strength: 19 (kgf)Control: handgrip strength: 21 (kgf)
Brown *et al*^31^	2020	ConHD: grip strength: 128.7±29.8 (N), bicep strength: 223.1 (N), quad strength: 618 (N)	ConHD: grip strength: 123.8±37 (N), bicep strength: 191.5 (N), quad strength: 567.3 (N)
Brassard *et al*^33^	2006	ConHD: MVC of the non-dominant arm muscles: 25.1±11.3 (kg)ConHD: MVC of the quadriceps femoris: 29.8±8.3 (kg)	ConHD: MVC of the non-dominant arm muscles: 29.1±8.4 (kg)ConHD: MVC of the quadriceps femoris: 27.1±6.1 (kg)
		**Adverse events**	
Cordina *et al*^13^	2013	Transient ischaemic (n=1)	
Sutherland *et al*^36^	2018	Arrhythmia (n=2)	
Aronoff *et al*^39^	2023	Intercurrent illness (n=1), medically unstable (n=1)	

If CON data are absent in the respective study, then not reported

CPET, cardiopulmonary exercise test; FEV1, forced expiratory volume; FVC, forced vital capacity; HR, heart rate; IMT, inspiratory muscle training; MEP, maximal expiratory pressure; MIP, maximal inspiratory pressure; MVC, maximum voluntary contraction; N/A, not applicable; VE, ventilation volume; VE/VCO_2_, ventilatory equivalent for carbon dioxide; V̇O_2_, oxygen consumption.

#### Peak oxygen consumption

**Figure 2:**
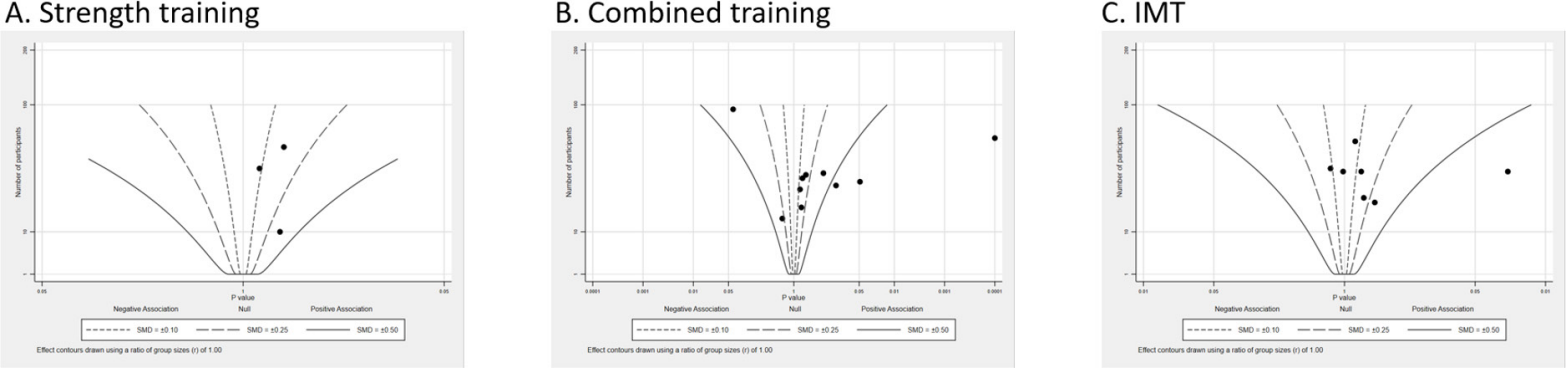
Albatross plot (peak oxygen consumption), subgroup by intervention. IMT, inspiratory muscle training; SMD, standardised mean difference.

20 studies examined CRF by measuring peak V̇O_2_ scaled to body mass in 338 participants with ConHD^1011 13 14 24^,^27 30 32 33 36^ (table 3). 16 studies observed a small improvement in peak V̇O_2_, with the points mostly clustered around the 0.10 to 0.50 SMD contours, indicating a small to moderate effect (figure 2). However, heterogeneity among these studies is demonstrated, with sample sizes ranging from 10 to 100, and the p values were spread horizontally along figure 2.

##### Subgroup by different interventions

All three studies from ST alone observed trivial to small improvements in peak V̇O_2_, with SMD values in the positive range of approximately 0.10 to 0.25 (figure 2A). Eight studies of combined training were centred to the right of the SMD contour (figure 2B), with four studies suggesting a trivial to small effect (0.1–0.25 SMDs). Other combined training studies were more dispersed and heterogeneous. For IMT intervention, four studies were clustered around the SMDs in the range of a trivial to small effect of 0.1 to 0.25 (figure 2C).

##### Subgroup by different age

**Figure 3:**
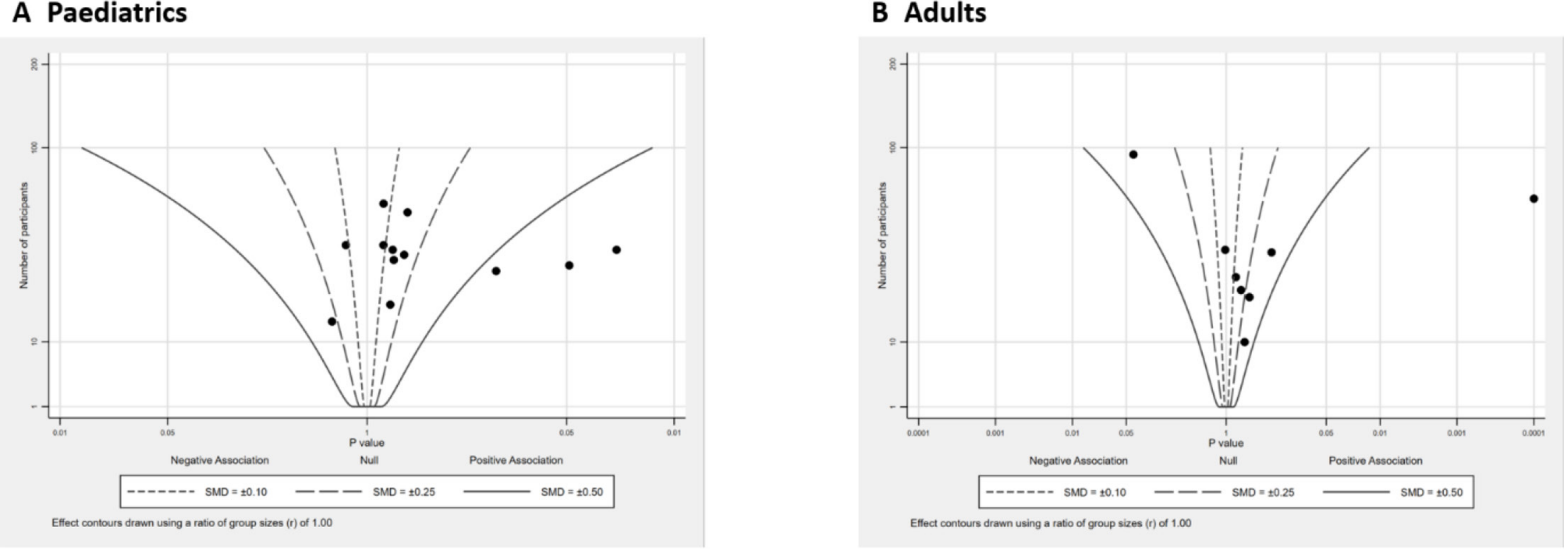
Albatross plot (peak oxygen consumption), subgroup by age. SMD, standardised mean difference.

Age-based analysis revealed a small improvement in peak V̇O_2_ (0.10–0.25 SMD) in 7 of 12 studies on paediatric participants (figure 3A), while 3 studies showed a positive effect (>0.50 SMD). Two studies showed a negative effect around −0.10 to −0.25 SMD contours, indicating a detriment in performance. Five studies showed small to moderate improvements (0.10–0.50 SMD) in peak V̇O_2_ in adults (figure 3B), with only one study showing no change in peak V̇O_2_ preintervention and postintervention.

##### Subgroup by different ConHD types

**Figure 4:**
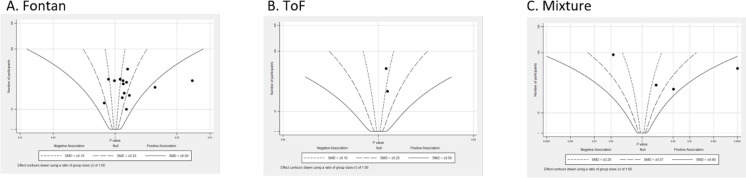
Albatross plot (peak oxygen consumption), subgroup by type of ConHD. SMD, standardised mean difference; ToF, tetralogy of Fallot.

A total of 14 studies were reported in Fontan patients (figure 4A). Eleven studies were centred to the right of the effect size contour, with the SMDs in the range of 0.1 to 0.5. All two studies reported a trivial improvement in people with ToF. Four studies reported higher heterogeneity in participants with a mixture of participants with ConHD.

### Pulmonary function

**Figure 5:**
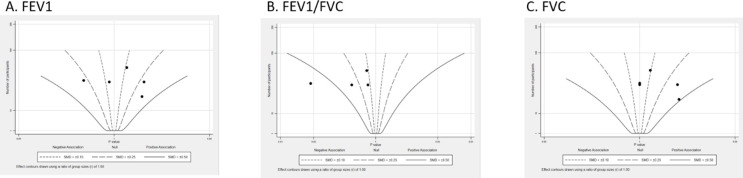
Albatross plot (pulmonary function), subgroup by forced expiratory volume in the first second (FEV1), forced vital capacity (FVC) and FEV1/FVC.

Only five IMT intervention studies involved pulmonary function outcomes (figure 5). Studies were mainly distributed around different SMD contours. For forced expiratory volume in the first second (FEV1), three studies had positive effects with the SMDs in the range of 0.1–0.25 (figure 5A). FEV1/FVC were negatively impacted across four studies reporting that the SMDs were in the range of −0.11 to −0.50 (figure 5B). FVC showed positive effects in three studies with SMDs between 0.10 and 0.50, while two studies reported null p values (figure 5C).

### Adverse events

11 studies (388 participants) reported adverse events,^13 24 25 30 31 33 34 36 39 42 44^ of which 8 studies reported no adverse events and three studies reported a total of 5 participants with adverse events.^13 36 39^ Of the five adverse events, two patients exhibited arrhythmia and systemic saturation <80% during combined training (aerobic and ST). One patient displayed an intercurrent illness and one experienced pain and safety concerns related to an acute abdominal process as rehabilitation progressed (combined training). Another participant experienced a transient ischaemic episode over a weekend, 3 days after the most recent ST session (ST alone). There were no reported serious adverse events or fatalities.

### Other secondary outcomes

For body composition, health-related quality of life, PA and other cardiorespiratory fitness outcomes other than peak V̇O_2_, we did not include them in the systematic review results due to insufficient data in the included literature and a lack of standardised study outcome measures.

### Risk of bias analysis

The risk of bias assessment details for CRF, pulmonary function and muscular strength are in online supplemental figures. Among 22 studies reporting CRF, 2 (9%) were assessed as ‘low risk’, 13 (59%) as ‘some concerns’ and 7 (32%) as ‘high risk’. For muscle strength, two (40%) had ‘some concerns’ and three (60%) were ‘high risk’. In five pulmonary function studies, one (20%) was ‘low risk’, four (80%) ‘some concerns’ and none ‘high risk’.

### The certainty of the evidence

For ST compared with usual care or no training, the evidence was very low-certainty for muscle strength, low-certainty for peak V̇O_2_ and pulmonary function, and moderate-certainty for adverse events (online supplemental table).

## Discussion

This review shows that ST interventions either as ST alone or combined training, or IMT may have a small beneficial effect on CRF, muscle strength and pulmonary function based on low-certainty to very low-certainty evidence and the paucity of ST alone studies. It is noteworthy that among the 20 studies, 16 studies indicated an improvement in peak V̇O_2_, although with a degree of heterogeneity, most likely due to the mode of combined training and an emphasis on aerobic exercises. Muscle strength was found to increase in three of five ST studies. Encouragingly, adverse events were infrequent or of minor severity in these studies. Notably, there were no serious adverse events or deaths associated with ST interventions.

Across five different studies (two ST alone, one combined, two IMT), two studies demonstrated statistically significant improvements in muscle strength across various anatomical locations in people with ConHD after ST alone. Conversely, the discernible impact of IMT and combined training on muscle strength appeared less pronounced. These findings still indicate that ST alone was more effective in augmenting peripheral muscle strength. As in our study, a meta-analysis investigating ST alone effects in people with acquired cardiac failure found significantly increased MVC (SMD: 0.76 (95% CI 0.26, 1.25), p=0.003) compared with usual care. While our findings suggest that ST has the potential to improve peripheral muscle strength in people with ConHD, the generalisability of these results remains uncertain. Notably, four out of five studies included in this review focused exclusively on Fontan patients, who represent a specific subgroup within the broader ConHD population. Given that Fontan physiology is characterised by unique haemodynamic and circulatory adaptations, the observed improvements in muscle strength may not be directly applicable to individuals with other forms of ConHD. Furthermore, three of the five studies demonstrated a serious risk of bias. This raises concerns regarding the robustness of the reported effects of ST. Given these constraints, further well-controlled trials with larger, more diverse ConHD cohorts are needed to confirm the efficacy of ST across different subtypes of ConHD.

This review could not establish an association between the effects of ST and the duration or intensity of the training regimen. We observed a general trend that emerged where any ST intervention surpassed an 8-week duration and exhibited positive changes for participants. The heterogeneity observed in these diverse training programmes precludes definitive conclusions regarding the optimal intensity and duration of training.

Peak V̇O_2_ represents the gold standard for assessing exercise tolerance and is commonly assessed for patients with ConHD.^5^ Within the scope of this present review, 16 of the 20 studies documented improvements in peak V̇O_2_, exhibiting a small effect size ranging from 0.10 to 0.50 SMD. Subgroup analysis of varied interventions revealed that in comparison to combined training and IMT, ST alone exhibited no significant positive effect on CRF. This outcome might be ascribed to the training intensity of 60 to 85% MVC observed in three studies focusing on ST interventions, which may indicate that high-intensity ST could intensify peak V̇O_2_ adaptations. It is noteworthy that exercise intensities within the range of 70–90% MVC have been identified as optimal for augmenting muscle mass in healthy people.^45^ Another rationale for the heightened enhancements associated with high-intensity ST resides in its capacity to bring individuals closer to their peak V̇O, consequently leading to a pronounced elevation in peak V̇O_2_.^45^ Nevertheless, the absence of a significant increase in peak V̇O_2_ may be attributed to the hypothesis that vascular adaptations may not occur until discernible alterations in muscle mass appear.^46^ Hence, the high intensity of ST while increasing the volume of repetitions may potentially yield superior CRF benefits, but the optimal intensity remains uncertain. The findings regarding the impact of combined training and IMT on CRF align with conclusions drawn from prior systematic reviews focusing on PA interventions for people with ConHD. The review concluded maximal CRF increased by a mean difference of 1.89 mL/kg/min (95% CI −0.22 to 3.99).^5^

Pulmonary functions derived from IMT interventions revealed enhanced FVC among patients, while FEV1 and FEV1/FVC showed non-significant improvements. The absence of significant changes in FEV1 and FEV1/FVC could potentially be attributed to the baseline levels of pulmonary function, which might have constrained the capacity for further enhancement through IMT, the baseline FEV1/FVC ratios ranged from 80.6% to 87.8%, and the scope for additional improvements in pulmonary function and pulmonary blood flow via IMT might have been limited. Furthermore, the pulmonary function outcomes of this review align with those of a prior systematic review of IMT interventions in people with chronic heart failure,^47^ indicating that the beneficial impacts of IMT on people with ConHD may operate independently of discernible alterations in pulmonary function.

In the trials where adverse events were documented, three adverse events were reported among 388 participants. No occurrences of sudden cardiac death were reported in any of the studies. Furthermore, none of the studies incorporating high-intensity ST documented injuries, increased blood pressure, diminished cardiac output or occurrences of bradycardia among the participants. This observation indicates that adherence to standardised protocols can mitigate potential risk factors associated with high-intensity ST for people with ConHD. Cordina *et al*^13^ documented an instance of transient ischaemic episodes after ST. Although the risk of bleeding related to exertion during ST was deemed minimal, this adverse incident might be associated with high-intensity ST. In contrast, Scheffer *et al*^14^ reported no adverse events, showing that a progressive increment in the intensity of ST interventions could potentially enhance participants’ physical adaptability, reducing the likelihood of adverse events.

The overall quality appraisal of the literature exhibited a range from very low to moderate quality. Specifically, the absence of adequate statistical plans and publicly available data in the registered protocols among most studies reported undermines their interpretations. This absence of detailed protocols raises further concerns regarding the robustness of data processing and analytical methodologies used, thereby increasing the susceptibility to contentious discussions and interpretations.

### Limitations

The limitation of this review predominantly stems from the lack of studies focusing on ST interventions among people with ConHD. The existing research also lacks a detailed description of the characteristics inherent in ST interventions, including frequency, intensity, duration and specific exercise types. These gaps highlight the need for future research to adopt standardised and clinically relevant outcome measures, ensuring more comprehensive and comparable findings. Additionally, longitudinal studies with larger sample sizes are necessary to evaluate the long-term effects of strength training on both physiological and psychosocial well-being in individuals with congenital heart disease, ultimately providing stronger evidence to guide clinical practice and rehabilitation strategies.

## Conclusion

This review systematically examines the evidence on the effects of ST on muscle strength, CRF and pulmonary function in people with ConHD. The findings show that ST alone can improve muscle strength. However, given that the majority of included studies focused on Fontan patients, the extent to which these benefits apply to the broader ConHD population remains uncertain. Both ST and combination training, as well as IMT, have a potential small improvement in peak V̇O_2_. However, discernible evidence supporting the enhancement of pulmonary function through ST is still lacking. The review highlights the safety of incorporating ST for individuals with ConHD; more studies should be required to explicitly report on adverse events, even if none are present. Future RCTs and long-term follow-up studies should improve their methodologies to validate the impact of ST on ConHD.

## Supplementary material

10.1136/openhrt-2024-003091 online supplemental file 1

10.1136/openhrt-2024-003091 online supplemental file 2

10.1136/openhrt-2024-003091 online supplemental file 3

## Data Availability

No data are available.
