# Supplementary material for: The effect of strength training interventions on people with congenital heart disease: a systematic review
Source: Open Heart. 2025 Mar 25;12(1):e003091. doi: 10.1136/openhrt-2024-003091 (PMC11938245; doi:10.1136/openhrt-2024-003091)
Supplement: online supplemental file 1 [file openhrt-12-1-s001.docx]

| **Search strategy**  **The Effect of Strength Training Interventions on People with Congenital Heart Disease: A Systematic Review**   - MEDLINE Ovid (137) - SPORTDiscus (58) - Web of science (1682) - Scopus (11) - Embase (259) - Cochrane Library (403) |
| --- |
| **Medline**  1 congenital heart disease/  2 (CHD* or ConHD or congenital or Fontan or Fallot).tw,kw.  3 1 or 2  4 strength training/  5 (strength train* or resistance train* or weight train* or inspiratory muscle train*).tw,kw.  6 (rehabilitation or exercise NEAR/3 (program* or train*)).tw,kw.  7 4 or 5 or 6  8 3 and 7  **Embase**  #1. 'congenital heart disease'/mj  #2. CHD*:ti,kw OR Fontan:ti,kw OR Fallot:ti,kw OR congenital OR ConHD:ti,kw  #3. #1 OR #2  #4. 'strength training'/mj  #5. (strength train* OR resistance train* OR weight train* OR inspiratory muscle train*):ti,ab,kw  #6. (rehabilitation OR exercise NEAR/3 (program* OR train*)).tw,kw.  #7. #4 OR #5 OR #6  #8. #3 AND #7  **Cochrane Library**  #1. [mh ^congenital heart disease]  #2. (CHD* OR congenital OR Fontan OR Fallot OR ConHD):ti,ab,kw  #3. {OR #1-#2}  #4. [mh ^“strength training”]  #5. (strength train* OR resistance train* OR weight train* OR inspiratory muscle train*):ti,ab,kw  #6. (rehabilitation OR exercise NEAR/3 (program* OR train*)).tw,kw.  #7. {OR #4-#6}  #8. #3 AND #7  **SPORTDiscus**  S1. SU congenital heart disease  S2. TI (CHD* or congenital or Fallot or Fontan or ConHD)  S3. S1 OR S2  S4. SU strength training  S5. TI ((strength train* or resistance train* or weight train* or inspiratory muscle train*)) OR AB ((strength train* or resistance train* or weight train* or inspiratory muscle train*))  S6. TI ((rehabilitation or exercise NEAR/3 (program* or train*)) OR AB ((rehabilitation or exercise NEAR/3 (program* or train*)))  S7. S4 OR S5 ORS6  S8. S3 AND S7 [Narrow by Language: - english]  **Web of science**  #1. TS congenital heart disease  #2. (TI=(CHD* or congenital or Fallot or Fontan or ConHD))) OR (AB=(CHD* or congenital or Fallot or Fontan or ConHD)))  #3. #1 OR #2  #4. TS strength training  #5. (TI=(strength train* or resistance train* or weight train* or inspiratory muscle train*))) OR (AB=(strength train* or resistance train* or weight train* or inspiratory muscle train*)))  #6. (TI=(rehabilitation or exercise NEAR/3 (program* or train*))) OR (AB=(rehabilitation or exercise NEAR/3 (program* or train*)))  #7. #4 OR #5 OR #6  #7. #3 AND #7 and Articles (Document Types) and English (Languages)  **Scopus**   1. TITLE(“congenital heart disease”) 2. TITLE-ABS(CHD* or congenital or Fallot or Fontant* or ConHD) 3. 1 OR 2 4. TITLE(“strength training”) 5. TITLE-ABS(strength train* or resistance train* or weight train* or inspiratory muscle train*) 6. TITLE-ABS(rehabilitation or exercise NEAR/3 (program* or train*) 7. 4 OR 5 OR 6 8. 3 AND 7 ( LIMIT-TO ( LANGUAGE,"English" ) ) |
