# Supplementary material for: The effect of strength training interventions on people with congenital heart disease: a systematic review
Source: Open Heart. 2025 Mar 25;12(1):e003091. doi: 10.1136/openhrt-2024-003091 (PMC11938245; doi:10.1136/openhrt-2024-003091)
Supplement: online supplemental file 2 [file openhrt-12-1-s002.docx]

Table S1. Summary of findings for the main comparison. Strength training compared to usual care or none for people with congenital heart disease

**Author(s):** Kunyu Hao

**Question:** Strength training compared to Usual care or None for people with congenital heart disease

**Setting:** hospital-based and home-based settings

**Bibliography:**

| **Certainty assessment** | | | | | | | **Impact** | **Certainty** | **Importance** |
| --- | --- | --- | --- | --- | --- | --- | --- | --- | --- |
| **№ of studies** | **Study design** | **Risk of bias** | **Inconsistency** | **Indirectness** | **Imprecision** | **Other considerations** |  |  |  |
| **Muscle strength (assessed with: dynamometer)** | | | | | | | | | |
| 2 | Randomised controlled trials | serious^a^ | serious^b^ | serious^c^ | serious^d^ | none | The outcomes of the two studies were not identical, possibly due to differences in the interventions: one study was a high-intensity resistance training intervention, and the other was an IMT. In addition, the parts of muscle strength measured are not the same. One study measured the strength of bending and stretching in different parts of the body. The other measured only grip strength. | ⨁◯◯◯ Very low | CRITICAL |
| **Cardiorespiratory fitness (assessed with**: **peak V̇O_2_**) | | | | | | | | | |
| 7 | Randomised controlled trials | serious^a,e,f,g^ | not serious^h^ | not serious | serious^d^ | none | 15 of 19 studies observed an improvement in peak V̇O_2_, with SMD from 0.1 to 0.5. | ⨁⨁◯◯ Low | CRITICAL |
| **Pulmonary function (assessed with: FEV1 and FVC)** | | | | | | | | | |
| 4 | Randomised controlled trials | serious^a,e,f,g^ | not serious^i^ | not serious | serious^d^ | none | Only the IMT studies involving pulmonary function outcomes. IMT interventions may non-significant improvements. | ⨁⨁◯◯ Low | CRITICAL |
| **Muscle strength (assessed with: dynamometer)** | | | | | | | | | |
| 3 | non-randomised studies | very serious^j,k,l^ | not serious | not serious | serious^d^ | none | The outcomes of the three studies were improved. | ⨁◯◯◯ Very low | CRITICAL |
| **Cardiorespiratory fitness (CRF) (assessed with: peak V̇O_2_)** | | | | | | | | | |
| 15 | non-randomised studies | serious^j,k,l^ | not serious | not serious | serious^d^ | none | 15 of 19 studies observed an improvement in peak V̇O_2_, with SMD from 0.1 to 0.5. | ⨁⨁◯◯ Low | CRITICAL |
| **Pulmonary function (assessed with: FEV1 and FVC)** | | | | | | | | | |
| 1 | non-randomised studies | not serious | not serious | serious^m^ | serious^d^ | none | FEV1 decreased, FVC slightly increased, and FEV1/FVC decreased in this study, | ⨁⨁◯◯ Low | CRITICAL |
| **Advent events** | | | | | | | | | |
| 11 | non-randomised studies | not serious | not serious | not serious | not serious | publication bias suspected^n^ | A participant experienced a transient ischemic episode over the weekend, 3 days after his most recent training session. No serious adverse events or fatalities were reported during the intervention programme. | ⨁⨁⨁◯ Moderate | CRITICAL |

**CI:** confidence interval; IMT, inspiratory muscle training; peak V̇O_2_, peak oxygen consumption; SMD, standardized mean difference; FEV1, forced expiratory volume in the first second; FVC, Forced vital capacity.

#### Explanations

a. Potentially, the outcome assessors may have been aware of the intervention. But no information was reported in full text or protocol

b. two studies have different outcome in terms of muscle strength. One study showed muscle strength significantly increased in different body parts but another showed no change in grip strength.

c. Indirectness occurs when the outcome studied is a surrogate for a different outcome. There is an IMT intervention and it measures handgrip this seems like indirectness.

d. The simple size is <400.

e. There was no information on method of randomisation and no baseline imbalance that would suggest a problem with randomisation

f. Both participants and those delivering the intervention were aware of the intervention received. There was no information to support deviations from the intended intervention.

g. There is no information on whether there were multiple eligible analyses.

h. Some studies showed peak VO2 increased but some some studies showed peak VO2 no change

i. the pulmonary function outcomes were same: no change, a little increase or decrease.

j. the interventions were not well defined: included frequency, duration, intensity and time/

k. missing data due to more than 30% of participants dropping out.

l. knowledge may influence outcomes

m. Only one study reported pulmonary function.

n. Over 58% of studies did not report data on adverse events. Therefore, the certainty of evidence was downgraded by 1 level due to publication bias.
