# Supplementary material for: The effect of strength training interventions on people with congenital heart disease: a systematic review
Source: Open Heart. 2025 Mar 25;12(1):e003091. doi: 10.1136/openhrt-2024-003091 (PMC11938245; doi:10.1136/openhrt-2024-003091)

Supplemental Figure 1. Risk of bias assessment for cardiorespiratory fitness on randomised controlled trials.


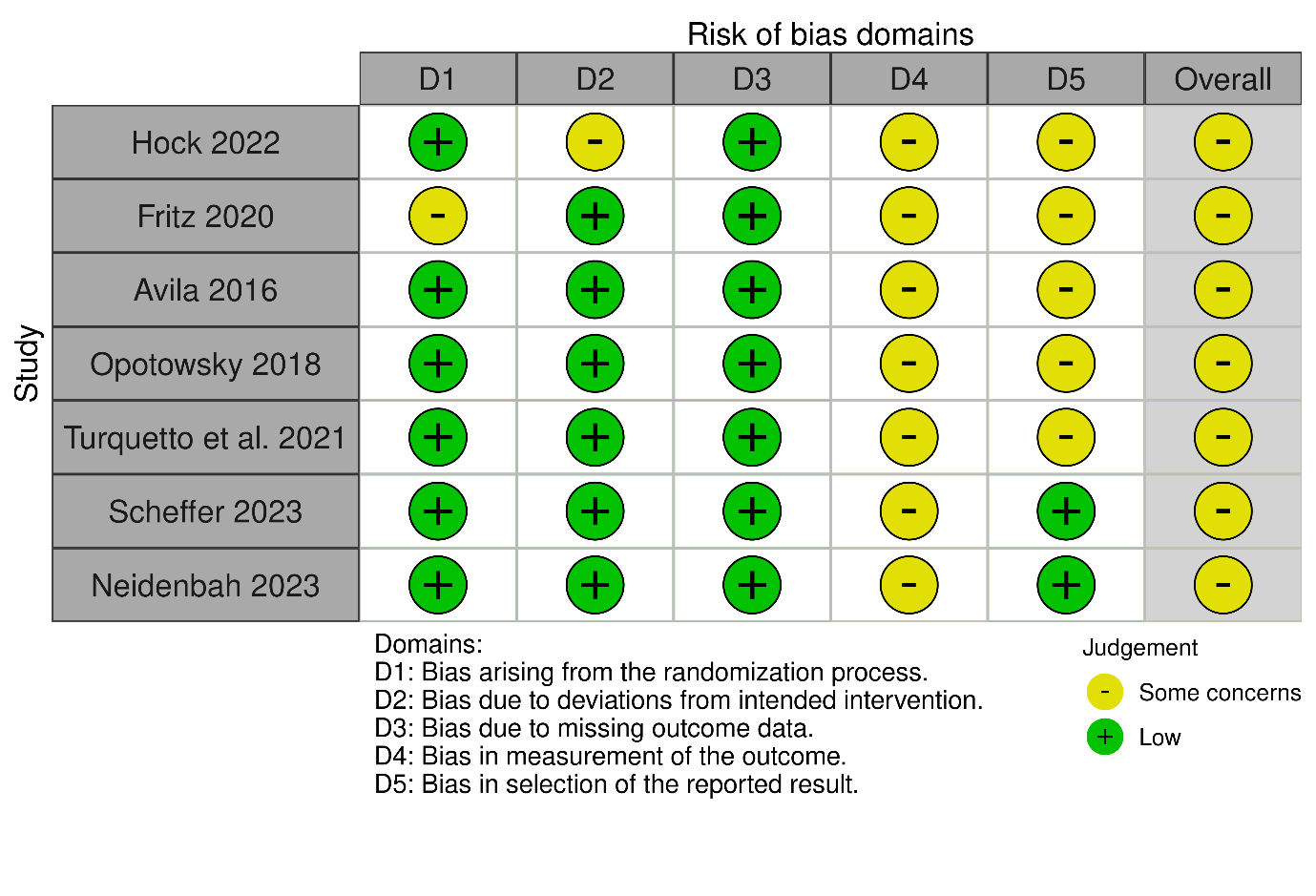


Supplemental Figure 2. Risk of bias assessment for muscle strength on randomised controlled trials.


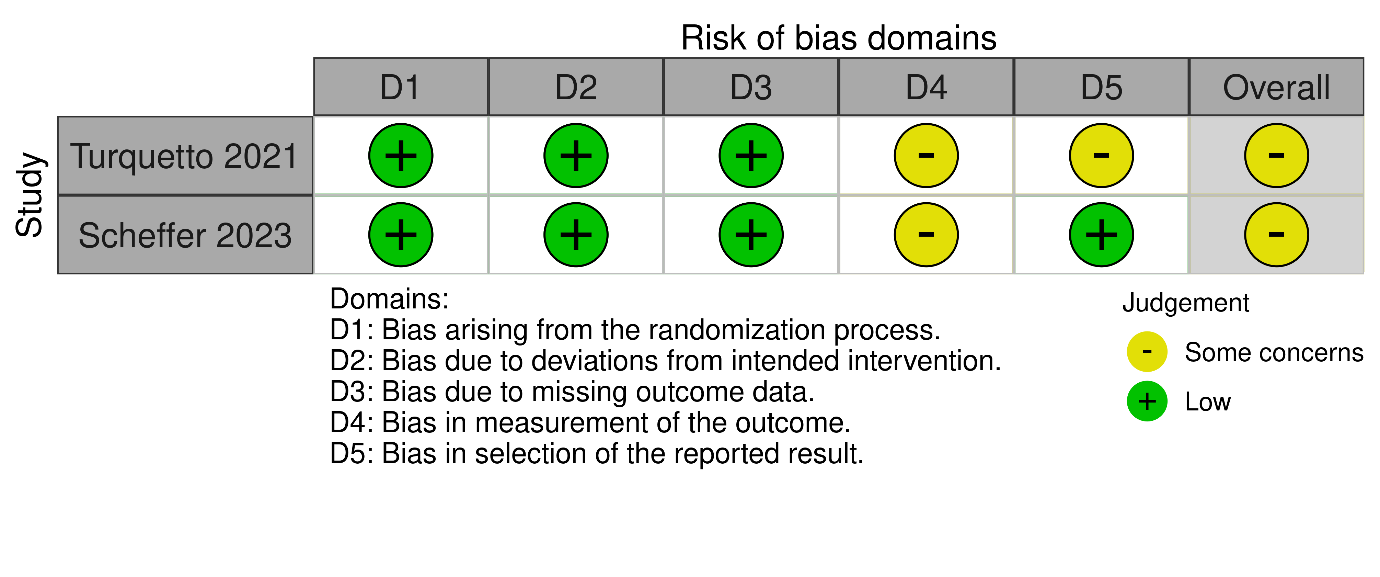


Supplemental Figure 3. Risk of bias assessment for pulmonary function on randomised controlled trials.


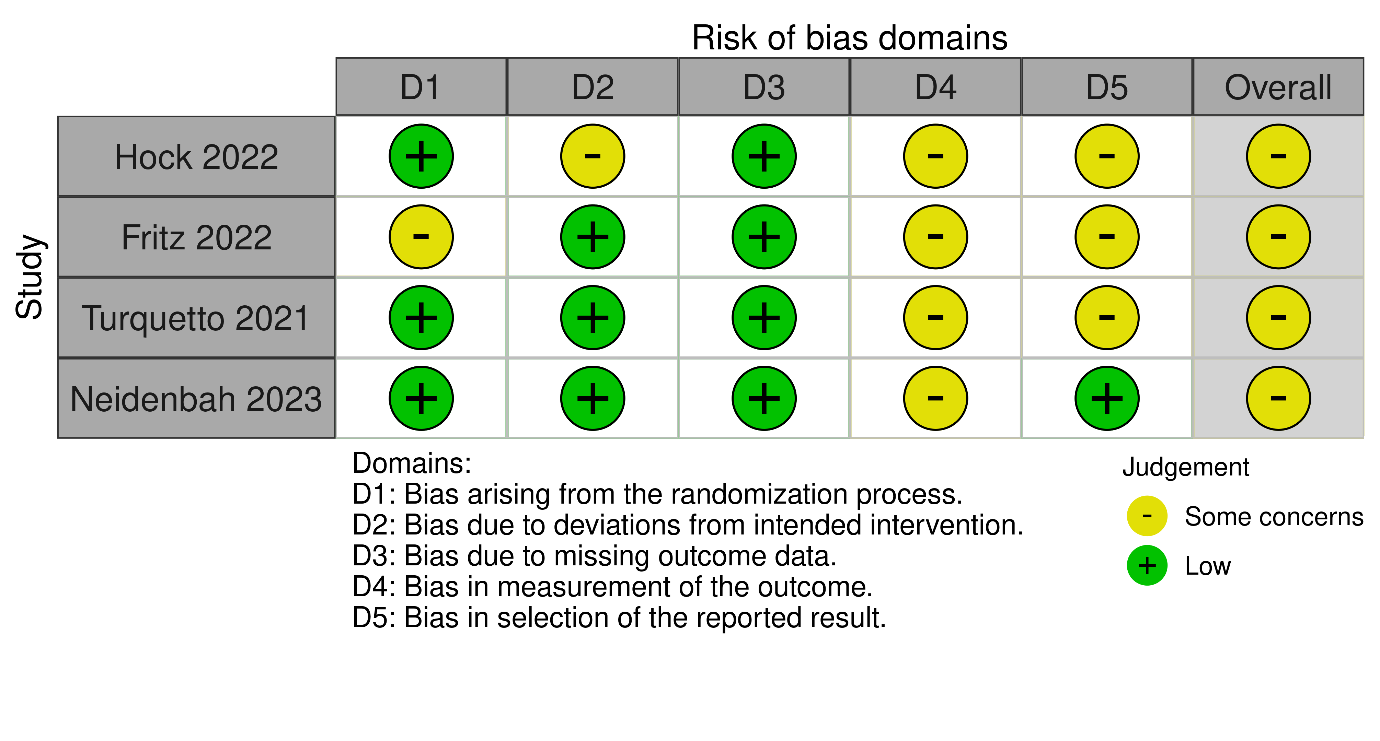


Supplemental Figure 4. Risk of bias assessment for cardiorespiratory fitness on non-randomised controlled trials and cohort studies.


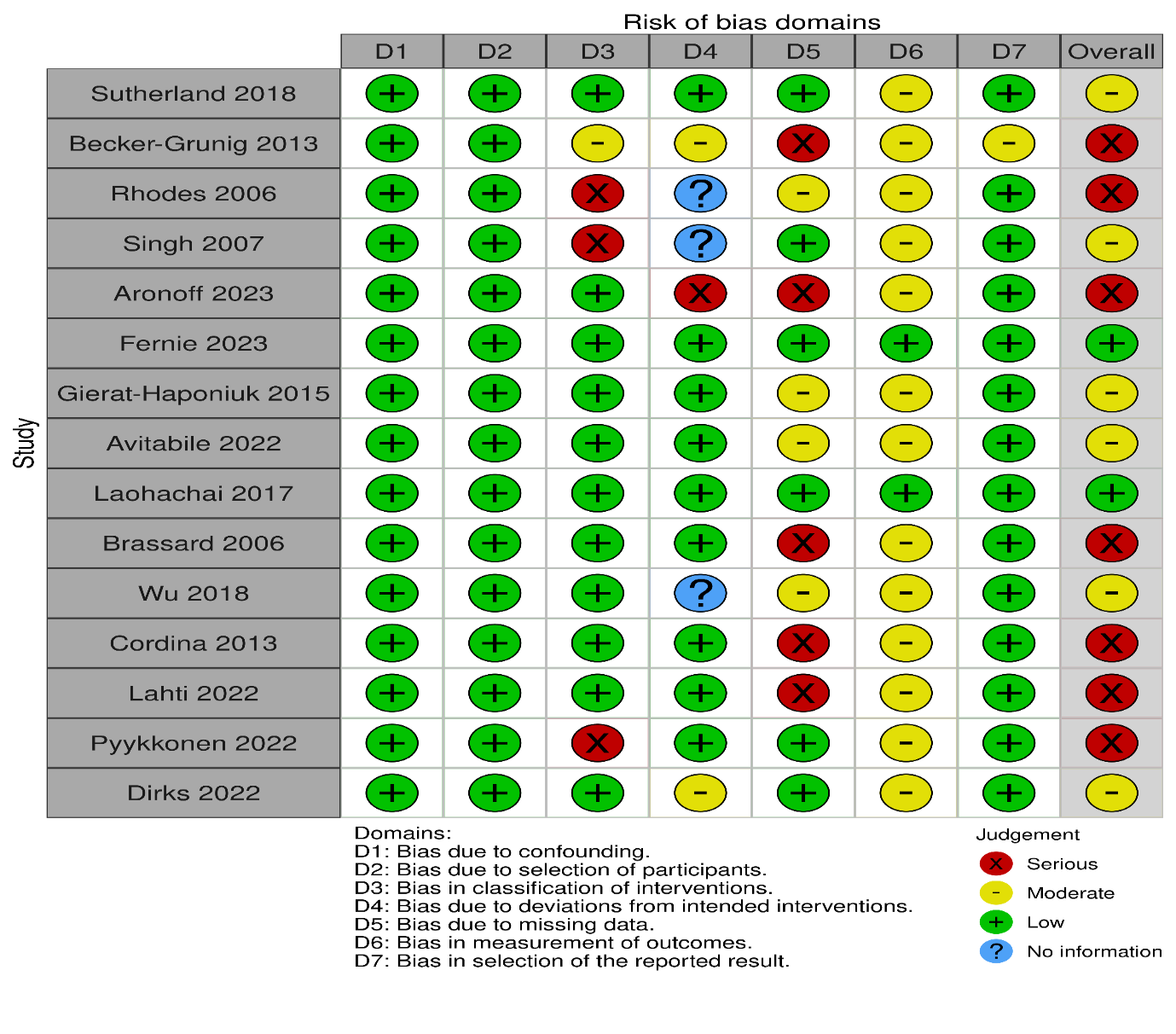


Supplemental Figure 5. Risk of bias assessment for muscle strength on non-randomised controlled trials and cohort studies.


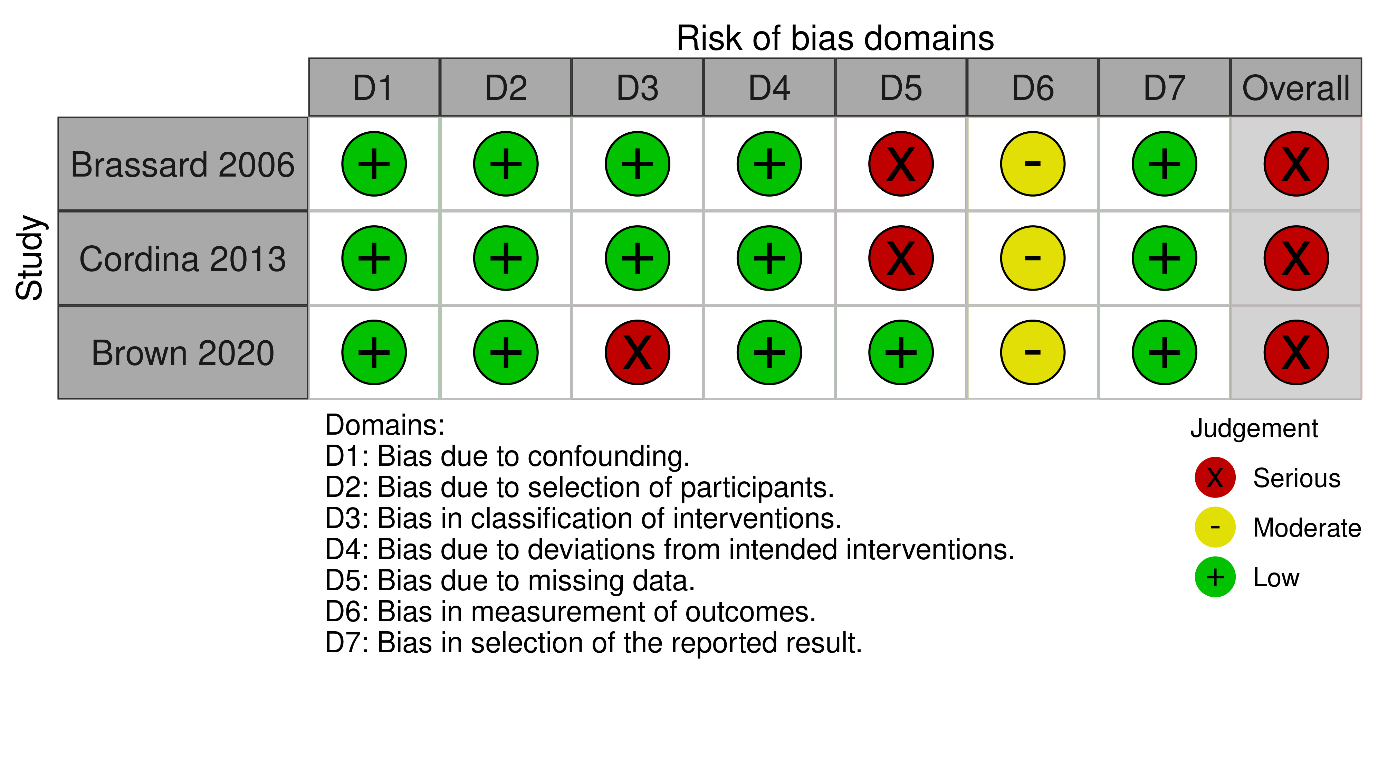


Supplemental Figure 6. Risk of bias assessment for pulmonary function on non-randomised controlled trials and cohort studies.


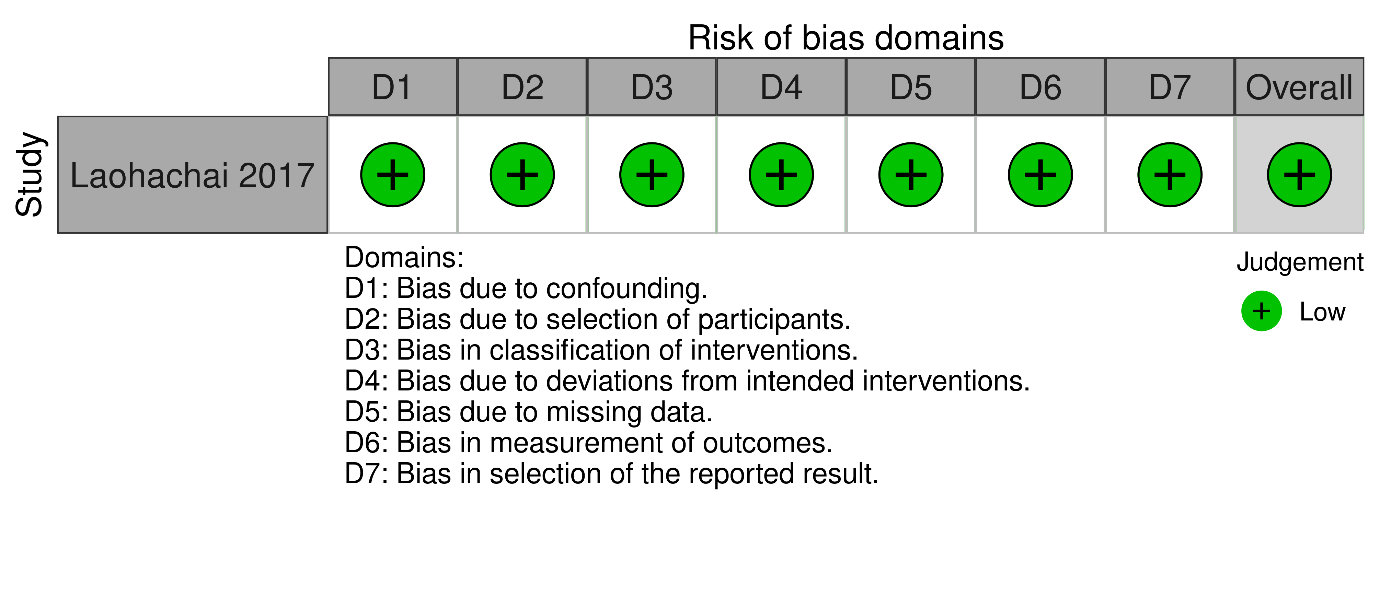

Supplement: online supplemental file 3 [file openhrt-12-1-s003.docx]
